# Adipokine expression and endothelial function in subclinical hypothyroidism rats

**DOI:** 10.1530/EC-18-0007

**Published:** 2018-01-12

**Authors:** Ningning Gong, Cuixia Gao, Xuedi Chen, Yu Wang, Limin Tian

**Affiliations:** 1 Department of EndocrinologyGansu Provincial Hospital, Lanzhou, China; 2 Department of Clinical MedicineGansu University of Chinese Medicine, Lanzhou, Gansu, China; 3 Department of Ultrasonic DiagnosisGansu Provincial Hospital, Lanzhou, China

**Keywords:** subclinical hypothyroidism, adipose tissue, adipokines, endothelial function, levothyroxine

## Abstract

The purpose of our study was to observe adipokine expression and endothelial function in subclinical hypothyroidism (sHT) rats and to determine whether levothyroxine (LT_4_) treatment affects these changes. Sixty-five male Wistar rats were randomly divided into five groups: the control group; sHT A, B and C groups and the sHT + T_4_ group. The sHT rats were induced by methimazole (MMI) and the sHT + T_4_ rats were administered LT_4_ treatment after 8 weeks of MMI administration. Thyroid function and lipid levels were measured using radioimmunoassays and enzymatic colorimetric methods, respectively. Serum adiponectin (APN), chemerin, TNF-α, endothelin (ET-1) and nitric oxide (NO) levels were measured using ELISA kits and a nitric-reductive assay. The expression of APN, chemerin and TNF-α in visceral adipose tissue (VAT) was measured in experimental rats using RT-PCR and Western blotting. Hematoxylin–eosin (HE) staining was used to observe changes in adipose tissue. The sHT rats had significantly higher levels of thyroid-stimulating hormone (TSH), TNF-α, chemerin, ET-1, total cholesterol (TC) and low-density lipoprotein cholesterol (LDL-C) and lower levels of APN and NO than those in control and sHT + T_4_ rats. Based on Pearson correlation analysis, the levels of chemerin, TNF-α, ET-1, LDL-C, TC and triglyceride (TG) were positively correlated with TSH, but APN and NO levels were negatively correlated with TSH. These findings demonstrated that high TSH levels contribute to the changes of adipokines and endothelial dysfunction in sHT, but LT_4_ treatment ameliorates those changes.

## Introduction

Subclinical hypothyroidism (sHT) is defined by increased thyroid-stimulating hormone (TSH) with normal thyroid hormone concentrations. The prevalence of sHT is 4–20% in adults (1). Recently, a relationship between sHT and atherosclerosis (AS) was reported in a large population-based study (2, 3). Multiple lines of evidence have shown that adipokines (4), endothelial dysfunction (5) and dyslipidemia (6) play a central role in the development of AS, but the relationship between these markers and TSH levels in the context of sHT is still unclear.

Adipose tissue is a major endocrine organ that secretes a variety of hormones and adipokines. TSH stimulates the production of adipokines in human abdominal adipose tissue and preadipocytes by affecting the function of the TSH receptor (TSHR) protein (7). Visceral adipose tissue (VAT) is the main adipose tissue that produces diverse adipokines, and VAT volume is increased in patients with sHT compared with VAT volume in normal controls (8). However, the morphological changes of adipocytes in sHT have not been studied.

Adiponectin (APN) is a recently described adipokine that plays a pivotal role in the inverse proportion to the degree of obesity and insulin resistance. Decreased levels of APN have a role in the pathology of AS in patients with hypothyroidism (9). Seifi and coworkers found that the mRNA levels of APN receptors are regulated by thyroid hormones in VAT of rats with hypothyroidism and hyperthyroidism (10). Chemerin, an 18-kDa protein secreted by adipose tissue, plays a key role in adipocyte differentiation and also helps to regulate APN expression and glycolipid metabolism (11). Several studies have demonstrated that chemerin is directly linked to human pathological states and increased insulin resistance and cardiovascular disease (CVD) (12, 13). In obesity, the expression of chemerin and its receptor are increased in VAT (14). TNF-α is also produced by adipose tissue as a pro-inflammatory adipokine associated with vascular endothelial diseases (15). The structure of TNF-α bears a marked similarity to the crystal structure of the globular domain of APN (16), and upregulated TNF-αs are associated with the effect of chemerin on CVD (17). Parlee and coworkers' (18) study also has shown that TNF-α may enhance prochemerin synthesis and secretion from adipocytes and TNF-α can regulate serum chemerin levels including increased secretion of prochemerin from intracellular stores and activation of serum proteases. It is an indirect effect. However, research to explore the changes of adipokines in sHT and the effect of LT_4_ treatment on these changes has been very limited.

Endothelial dysfunction is an early step of AS (19); it is related to decreased nitric oxide (NO) bioavailability and increased endothelin (ET-1) level. Some studies have shown that the TSH receptor (TSHR) is expressed in endothelial cells (20) and increases the risk of endothelial dysfunction (21). A recent study demonstrated that patients with sHT patients had significantly lower NO levels and a higher ET-1 levels than healthy people (5), but our previous clinical study showed no significant difference in serum ET-1 level between patients with sHT patients and euthyroid subjects (22). We speculated that these differences may be associated with TSH levels. Our present study aimed to explore changes of endothelial function in rats with sHT exhibiting different levels of TSH.

The relationship between APN, chemerin and TNF-α levels with endothelial function and the development of sHT with different TSH levels have not yet been investigated. Moreover, the effect of levothyroxine (LT_4_) treatment on adipokine levels and endothelial function is not yet clear. Our study aims to observe the changes in these adipokines and endothelial function in sHT rats and to verify the impact of LT_4_ on these indexes. In addition, lipids were also examined in our study as a risk factor for AS.

## Materials and methods

### Animal model

Sixty-five male Wistar rats were purchased from the College of Gansu Traditional Chinese Medicine Experimental Animal Center (Lanzhou, Gansu, China) and housed in a specific pathogen-free animal laboratory. After one week of environmental adaptation, rats were randomly divided into five groups: control euthyroid group (*n* = 10), sHT A group (*n* = 15), sHT B group (*n* = 15), sHT C group (*n* = 15) and sHT + T_4_ group (*n* = 10). In the sHT rats, groups A–C were separately induced by administration of 20 mg/kg/day methimazole (MMI), 30 mg/kg/day MMI and 40 mg/kg/day MMI once daily by gavage (13, 23, 24). The sHT + T_4_ group was administered 20 mg/kg/day MMI once daily by gavage. We measured serum T_4_ and TSH levels every 2 weeks. Control group rats were administered the same dose of normal saline. After 8 weeks, we measured increased levels of TSH and normal levels of T_4_ in sHT and sHT + T_4_ groups compared with the control group, confirming sHT induction. Subsequently, in sHT + T_4_ rats, we administered 6 mg/kg/day LT_4_ once daily by gavage (25, 26) at the same time as MMI administration until thyroid function was not significantly different from that of control rats. The body weight of rats was measured weekly.

### Drugs

MMI (Sigma) and LT_4_ (Merck KGaA DE-MRK) were dissolved in physiological saline, and their doses were tested in preliminary experiments.

### Serum specimen collection

The samples of abdominal aortic blood were collected in vacuum biochemical tubes. After incubation at room temperature for 30 min, the blood was centrifuged at 3000 ***g*** for 10 min at 4°C. The supernatant was transferred to separate tubes without disturbing blood clots and stored at −80°C.

### Adipose tissue specimen collection

The rats were anesthetized with 10% chloral hydrate by intraperitoneal injection. Animals were fixed on the operation panel, surgical scissors were inserted into inguinal areas of skin, and then visceral adipose tissue was clipped around the epididymis, placed in a frozen storage tube and immediately into liquid nitrogen, then transferred to a −80°C cryogenic refrigerator until use.

### Biochemical measurements

Serum TSH and T_4_ concentrations were measured using a radioactive immunity analysis method (North Biotechnology Research Co, Ltd, Beijing, China). Serum total cholesterol (TC), high-density lipoprotein cholesterol (HDL-C), low-density lipoprotein cholesterol (LDL-C) and triglyceride (TG) concentrations were measured by enzymatic colorimetric methods with assay kits (Sinopharm Chemical Reagent Beijing Co, Ltd., China). The serum NO concentration was measured using nitrate reductase and Griess reaction in frozen samples (Bioengineering Institute, Nanjing, China). Serum chemerin, APN, TNF-α and ET-1 concentrations were detected by ELISA kits (Enzyme-linked Biotechnology Co., Ltd., Shanghai, China).

### Real-time PCR

Total RNA was extracted using TRIzol (Invitrogen). Gene expression was detected by RT-PCR, 800 ng RNA samples were quantified by measuring the absorbance at 260 and 280 nm and then were reverse-transcribed to cDNA with a Bio-Rad iScript cDNA Synthesis Kit (Bio-Rad) in a reaction volume of 20 μL. The primers used were designed by Sangon Biotech (Shanghai, China). The RT-PCR protocol comprised 3 min of denaturation at 95°C, followed by 45 cycles of 95°C for 5 s, 60°C for 30 s and 72°C for 30 s using the iQ SYBR Green Supermix (Funglyn Biotech, Canada) in an FTC-3000 Fast Real-Time PCR System (Funglyn Biotech). Relative mRNA expression was calculated with the 2^−ΔΔCt^ method.

### Western blot

The VAT around the epididymis was ground on ice and incubated in RIPA buffer for 15–30 min. The tissue was ultrasonicated 4 times at 5 s each, and then centrifuged at 4°C and 10,000 ***g*** for 15 min. The supernatant was stored at −20°C. The proteins were separated by 15% SDS-PAGE and transferred to a 0.22-µm PVDF membrane. After blocking in 5% skim milk at room temperature for 2.5 h, the membrane was incubated overnight at 4°C in TBST with the following antibodies: anti-APN (Abcam) (1:1000), anti-chemerin (Abcam) (1:200), anti-TNF-α (ImmunoWay, USA) (1:1000) and GAPDH (ImmunoWay) (1:1000). The membrane was further incubated in secondary antibody (ZSGB BIO, Beijing, China) (1:5000) at room temperature for 2 h after washing three times with TBST. To visualize protein bands, we added a chemical luminescence reagent ECL (ImmunoWay) to the membrane for 1 min and developed it. Protein band intensities were expressed as a ratio to the intensity of the GAPDH band.

### Histopathology of the VAT

The VAT around the epididymis was harvested and fixed in 10% neutral buffer formalin at the end of the experiment. Sections of 5 μm were cut and stained with hematoxylin–eosin (HE, 20×) and visualized with a light microscope (Olympus BX 60).

### Ethics statement

Our study was carried out in strict accordance with the recommendations of the Guide for the Care and Use of Laboratory Animals by the National Institutes of Health. The protocol was approved by the Committee on the Ethics of Animal Experiments of the College of Gansu Traditional Chinese Medicine Experimental Animal Center. All operations were performed under sodium pentobarbital anesthesia, and all efforts were made to reduce the suffering of the experimental animals.

### Statistical analysis

Statistical analyses were performed with SPSS, version 19.0. For determining relative gene expression, the mean value of the control group rats was defined as 100%. Differences among groups were compared using one-way ANOVA. A *P* value of <0.05 was accepted as statistically significant. Numerical variables are shown as mean ± s.d. Pearson correlations were performed to verify the correlations between those biomarkers and TSH.

## Results

### Morphological changes

**Figure 1:**
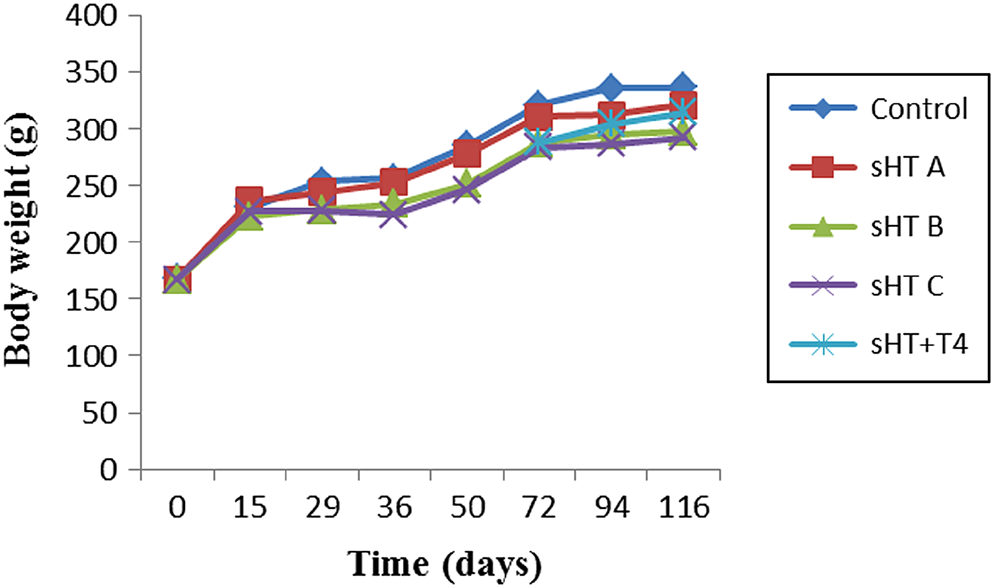
No significant differences were observed in body weights among the five groups.

At the end of the 4-month study period, the rats in the sHT group were less active and had drier than control rats. Rat body weights were decreased compared with those of the control group, but there was no statistical difference between any two groups (Fig. 1). Although body weights were improved in the sHT + T_4_ group compared with the sHT C group, there were no significant differences between these groups.

### Plasma thyroid hormone, TSH, lipids, NO, ET-1 and adipokines levels

There were no significant differences in the serum T_4_ levels observed among the five groups. Serum TSH levels in sHT groups (including sHT A, B and C) were significantly higher than levels in the control group (*P* < 0.05). Serum TSH in sHT + T_4_ group was significantly decreased compared with that of the sHT C group (*P* < 0.05) and still significantly higher than that of the control group (*P* < 0.05). Serum TC and LDL-C were related with the increase of TSH in sHT groups and LT_4_ treatment could decrease these changes in the sHT + T_4_ group (*P* < 0.05). The TG level was significantly increased in the sHT C group compared with the level in control and sHT + T_4_ groups (*P* < 0.05). Levels of chemerin and TNF-α in sHT groups were significantly higher than those of the control group, and those changes in the sHT + T_4_ group were significantly lower than those of the sHT C group, but still significantly higher than those of the control group (*P* < 0.05). However, serum APN levels were significantly decreased in sHT groups compared with levels in control and sHT + T_4_ groups (*P* < 0.05). Regarding endothelial function, the sHT rats displayed significantly decreased serum NO levels and an increased serum ET-1 levels compared with the levels observed in control rats (*P* < 0.05). LT_4_ treatment improved those changes in sHT + T_4_ rats (Table 1).

**Table 1:** The 5 groups were well matched in thyroid hormones, lipids and adipokines levels.

	Control group	sHT groups			sHT + T_4_ group
		A	B	C	
TT_4_ (ng/mL)	67 ± 6	61 ± 10	61 ± 9	60 ± 10	59 ± 5
TSH (µIU/mL)	0.42 ± 0.06	0.62 ± 0.07^a^	0.73 ± 0.07^a,b^	0.82 ± 0.07^a,b,c^	0.62 ± 0.08^a,d^
TC (mmol/L)	1.17 ± 0.72	1.66 ± 0.13^a^	1.81 ± 0.15^a,b^	2.4 ± 0.19^a,b,c^	1.67 ± 0.11^a,d^
LDL-C (mmol/L)	0.59 ± 0.15	0.88 ± 0.18^a^	0.91 ± 0.16^a^	1.15 ± 0.17^a,b,c^	0.92 ± 0.1^a,d^
TG (mmol/L)	0.43 ± 0.1	0.5 ± 0.13	0.56 ± 0.08	0.62 ± 0.15^a,b^	0.49 ± 0.11^d^
HDL-C (mmol/L)	0.86 ± 0.11	0.81 ± 0.12	0.84 ± 0.14	0.78 ± 0.07	0.8 ± 0.07
APN (µg/L)	115 ± 4	77 ± 3^a^	68 ± 3^a,b^	59 ± 2^a,b,c^	102 ± 3^a,d^
Chemerin (pg/mL)	202 ± 17	314 ± 16^a^	355 ± 17^a,b^	365 ± 12^a,b^	260 ± 10^a,d^
TNF-α (ng/L)	143 ± 18	222 ± 14^a^	279 ± 13^a,b^	288 ± 16^a,b^	178 ± 10^a,d^
ET-1 (nmol/L)	95 ± 18	146 ± 14^a^	156 ± 13^a^	168 ± 23^a,b^	102 ± 14^d^
NO (µmol/L)	298 ± 19	261 ± 17^a^	227 ± 27^a,b^	218 ± 14^a,b^	250 ± 19^a,d^

^a^ *P* < 0.05 vs control group; ^b^ *P* < 0.05 vs sHT A group; ^c^ *P* < 0.05 vs sHT B group; ^d^ *P* < 0.05 vs sHT C group.

APN, adiponectin; ET-1, endothelin; HDL-C, high-density lipoprotein cholesterol; LDL-C, low-density lipoprotein cholesterol; NO, nitric oxide; TC, total cholesterol; TG, triglyceride; TSH, thyroid-stimulating hormone; TT_4_, total thyroxin.

### Pearson correlation analysis

Pearson correlations were performed to determine the correlations between TSH levels and various biomarkers. The results showed that the level of TSH was significantly positively correlated with levels of TC, LDL-C, TG, chemerin, TNF-α and ET-1 (*P* < 0.01), but significantly negatively correlated with levels of APN and NO (*P* < 0.01). There was no correlation between HDL-C and TSH (Table 2).

**Table 2:** Pearson correlation analysis of various variables.

	TSH	
	*r*	*P*
TC (mmol/L)	0.790	<0.01
LDL-C (mmol/L)	0.697	<0.01
TG (mmol/L)	0.485	<0.01
HDL-C (mmol/L)	−0.175	0.163
APN (µg/L)	−0.798	<0.01
Chemerin (pg/mL)	0.816	<0.01
TNF-α (ng/L)	0.818	<0.01
ET (nmol/L)	0.684	<0.01
NO (µmol/L)	−0.727	<0.01

APN, adiponectin; ET-1, endothelin; HDL-C, high-density lipoprotein cholesterol; LDL-C, low-density lipoprotein cholesterol; NO, nitric oxide; TC, total cholesterol; TG, triglyceride; TSH, thyroid-stimulating hormone.

### The expression of adipokines in VAT

**Figure 2:**
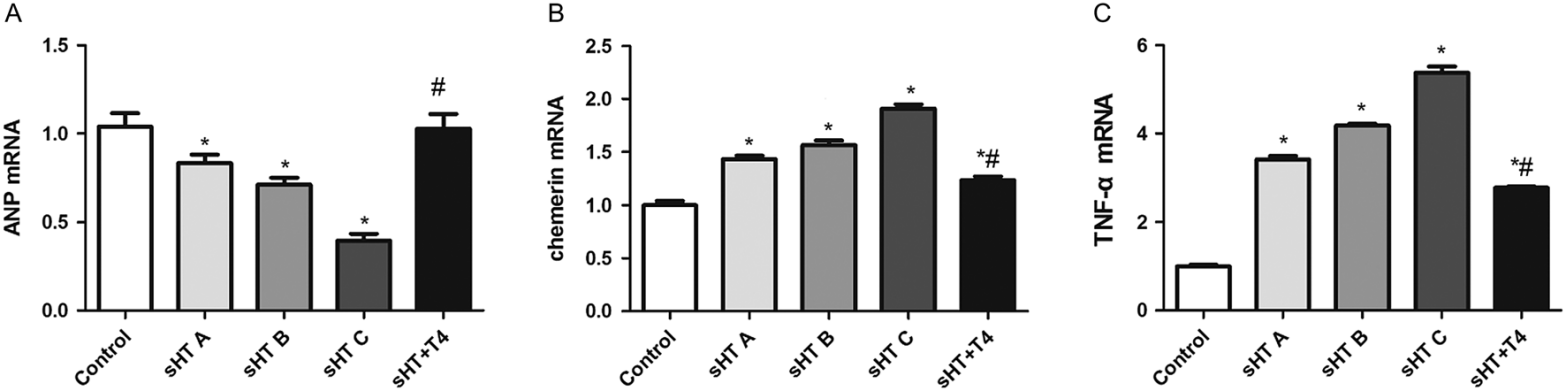
The expression of APN (A), chemerin (B) and TNF-α (C) mRNA in the VAT around the epididymis were measured with real-time PCR. *P < 0.01 vs control group; ^#^ *P* < 0.01 vs sHT C group.

Real-time PCR was used to measure the expression of APN, chemerin and TNF-α in VAT. As shown in Fig. 2, the expression of APN mRNA in sHT groups was significantly lower than that of the control group (*P* < 0.05); its level in the sHT + T_4_ group, which approached that of the control group, was significantly higher than that in the sHT C group (*P* < 0.05). The levels of chemerin and TNF-α mRNA were increased in sHT groups compared with those of the control group, but these levels were significantly decreased in the sHT + T_4_ group compared with those of the sHT C group (*P* < 0.05). From the sHT groups A to C, chemerin and TNF-α mRNA levels gradually increased and APN mRNA levels gradually decreased, there were significant differences between each group (*P* < 0.05).

**Figure 3:**
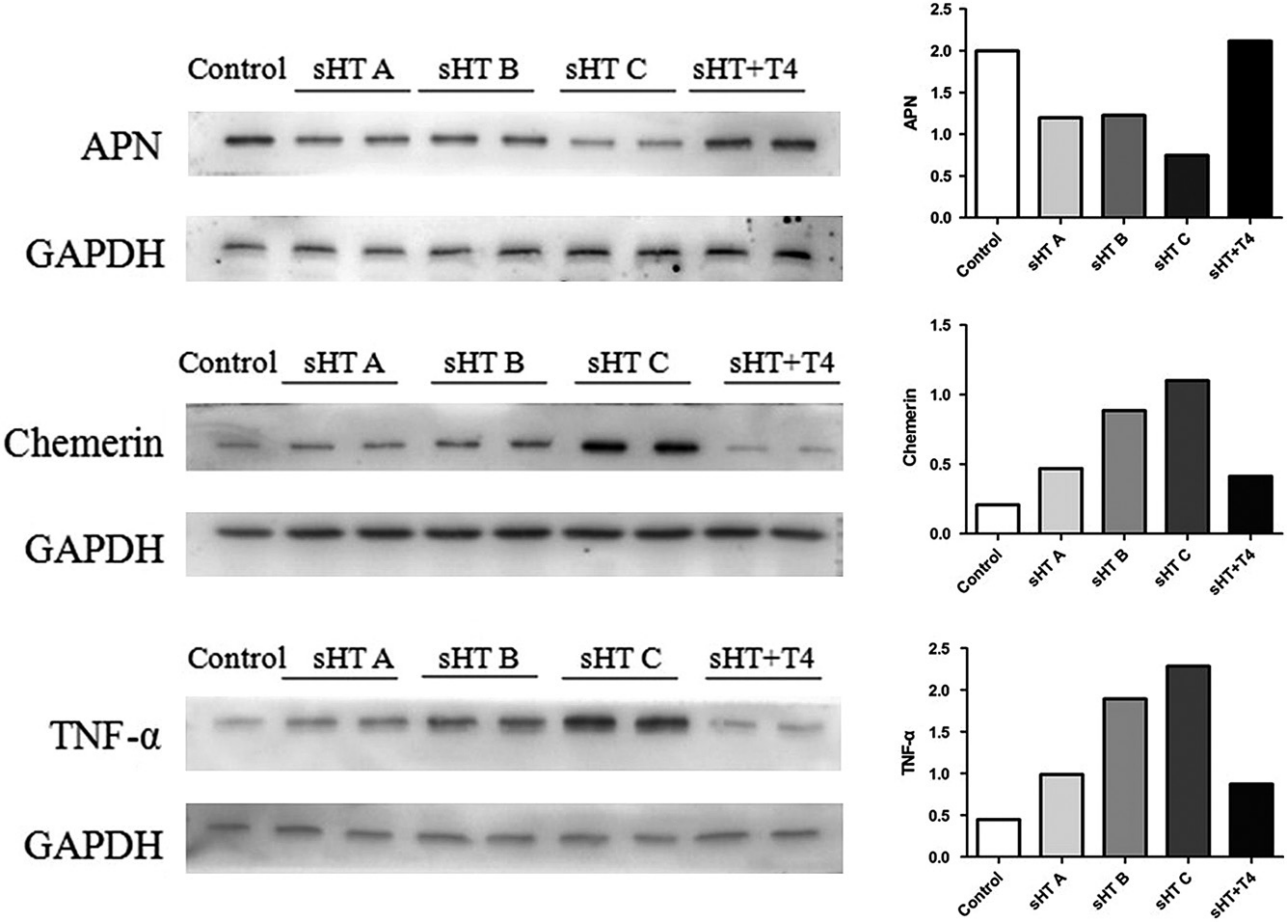
The protein expressions of APN, chemerin and TNF-α in the adipose tissue.

Western blots examining these adipokines’ protein levels are shown in Fig. 3. The sHT groups had significantly higher chemerin and TNF-α protein levels and a lower APN protein level than those of the control and sHT + T_4_ groups. From sHT groups A to C, the protein levels of chemerin and TNF-α were gradually increased. However, the APN protein level in the sHT C group was lower than that observed in the sHT A and B groups.

### HE staining

**Figure 4:**
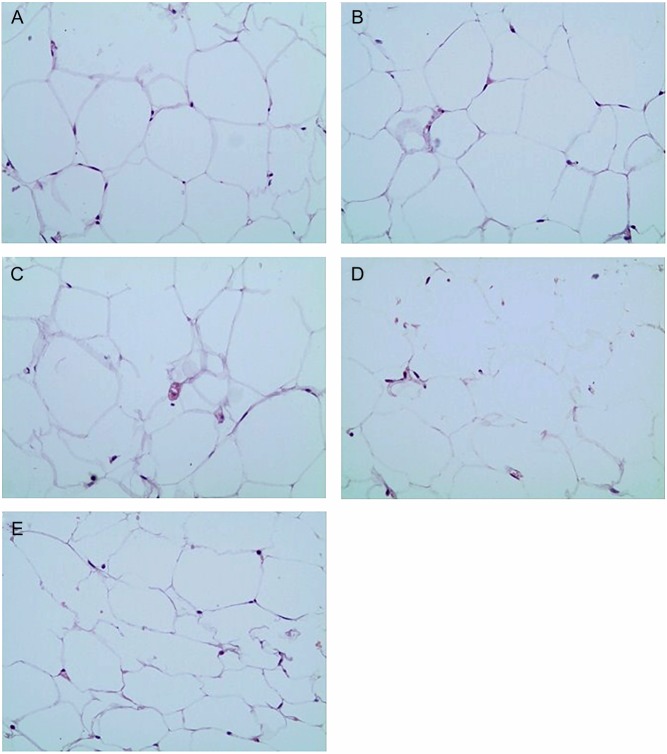
LT_4_ treatment ameliorated the size of adipose cells and arrangement. (A) Control group; (B) sHT A group; (C) sHT B group; (D) sHT C group; (E) sHT + T_4_ group.

As shown in Fig. 4, HE staining demonstrated that adipocytes were disordered and the adipocyte areas in sHT groups were larger compared with the adipocyte area in the control group. Compared with the sHT C group, LT_4_ treatment ameliorated these changes.

## Discussion

Thyroid dysfunction is a common endocrine disease and is accompanied by changes in intermediary metabolism. Clinical studies have suggested that the changes of some adipokines in thyroid dysfunction were associated with the development of AS (27). Endothelial dysfunction, an early step in AS development, is related to decrease NO bioavailability and ET-1 overexpression (28, 29). However, the relationship between changes in APN, chemerin, TNF-α and endothelial function with altered TSH levels are controversial and the relationships between those adipokines and endothelial function are still unclear in sHT.

We established a model of sHT in rats by gavage with MMI and treated one group of sHT rats with LT_4_. The dosages of MMI and LT_4_ were based on former studies (24, 25), and different doses of MMI have been verified and adjusted to establish a rat model of sHT in a 4-month experiment. We determined TSH and T_4_ levels to confirm that the sHT rat model was successful and that LT_4_ treatment was effective. After 8 weeks, the sHT rat model was successfully established. From sHT groups A to C, TSH levels gradually increased, and there were significant differences between each group after 16 weeks. Our experiment had a long time period to allow us to investigate the effects of different levels of TSH and the treatment of LT_4_ on the endothelial function, adipokines and lipids.

In our study, we concentrated on the changes of adipokines and endothelial function in sHT rats with different levels of TSH. Bell and coworkers found that the TSHR protein is also present in abdominal fat (30) and Iglesias and coworkers found a high expression level of TSHR in adipocytes (31), with functions similar to those in the thyroid (32). The expression of TSHR in adipocytes resulted in TSH-stimulating cAMP production (33). Arpaci and coworkers showed that increased VAT in sHT was associated with the development of AS (34). However, Yildiz and coworkers found that fat mass in sHT patients was not significantly altered (35), and the pathological changes of VAT are still unclear. In our study, we observed increased adipocyte size and inconsistent adipocyte morphology in VAT in animals with sHT, and these changes may result in adipose metabolism disorder by regulating TSH.

APN has anti-atherogenic and anti-inflammatory properties and increases insulin sensitivity, along with playing a protective role against AS and diabetes (36, 37). Seifi and coworkers found that APN receptor gene expression was decreased in VAT of rats with MMI-induced hypothyroidism compared to the expression in euthyroid rats (10). Studies by Cerbone and coworkers and Choi and coworkers showed that APN is a risk factor for CVD, but its level did not differ between patients with sHT and euthyroid controls (38, 39). Some studies demonstrated that serum APN levels in patients with sHT were not significantly altered, but presented significantly lower levels of APN than subclinical hyperthyroidism group (40). In our study, the expression of APN was significantly lower in sHT groups with the increased TSH levels, consistent with results of a few studies (41, 42). In addition, serum APN was significantly positively correlated with NO and negatively correlated with ET-1. These results may indicate that decreased APN is a risk factor in the development of endothelial dysfunction.

Chemerin, another adipokine secreted by adipose tissue, is linked to a series of metabolic disorders and increased risk of diabetes and CVD by upregulating TNF-α and IL-6 (17). The expression of chemerin and TNF-α were significantly higher with the increased TSH in VAT of sHT rats. Moreover, the expression of chemerin in experimental rats was correlated with increases in TNF-α (12, 18). There are few clinical studies that have examined changes of the above adipokines in sHT. Some studies have reported that chemerin and TNF-α levels were comparable between sHT and the control groups, but levels of these markers were significantly increased in hypothyroidism patients (43, 44). Here, we found that the levels of chemerin and TNF-α in sHT rats were significantly increased compared with those of control rats. Moreover, the levels of chemerin and TNF-α exhibited a positive relationship with TSH. The reason for these differences may be the differences in TSH levels. In addition, the levels of chemerin and TNF-α were positively related with ET-1 and negatively related with NO. Similarly, Gu and coworkers reported that chemerin was an independent predictor of impaired endothelial function (45).

Endothelial dysfunction is an early step in AS development, and ET-1 plays a key role in the process of endothelial dysfunction (46). Decreased circulating NO level is an early physiological event in AS and CVD (47). Our study has shown that rats with sHT displayed significantly decreased serum NO levels and increased serum ET-1 levels compared with those of control euthyroid rats. The level of ET-1 was significantly positively correlated with TSH, but the level of NO was significantly negatively related with TSH. This demonstrated that elevated TSH levels may contribute to AS through promoting impaired endothelial function by increasing ET-1 and decreasing NO levels (5).

We also established sHT + T_4_ rats to investigate whether LT_4_ treatment improved the observed changes. Recent clinical studies suggest that serum NO could be a reliable biomarker for the effect of T_4_ treatment in sHT (48). LT_4_ treatment has reversed endothelial dysfunction in patients with sHT (49). In the sHT + T_4_ group, serum NO was significantly higher and ET-1 was significantly lower than those observed in the sHT C group. Interestingly, some adipokines have been recommended as potential therapeutic targets for ameliorating AS development (50, 51). We observed increased APN expression and significantly decreased chemerin and TNF-α levels in LT_4_ treatment rats by examining gene and protein levels. Seifi and coworkers reported that APN mRNA was significantly increased in hypothyroid rats after 2 weeks of LT_4_ treatment. Yildiz and coworkers found that levels of APN and leptin were not different between sHT and controls (52), but replacement therapy significantly increased APN and decreased leptin levels, independent of changes in body fat mass (53). Hence, treatment with LT_4_ can ameliorate these changes in adipokine levels and endothelial function.

The well-known cardiovascular risk factor, serum lipid content, was also assessed to determine the risk of AS. Consistent with our previous research (54), we demonstrated that serum TC and LDL-C levels in the sHT group were significantly higher than those in control and LT_4_ treatment groups, but these and TG levels were positively correlated with TSH level.

In conclusion, our study demonstrated that sHT rats exhibited increased levels of ET-1, chemerin and TNF-α and decreased levels of NO and APN. Elevated TSH levels affected the expression of adipokines and the development of endothelial dysfunction. Moreover, the changes of those adipokines in sHT may be a risk factor for endothelial dysfunction. Fortunately, LT_4_ treatment ameliorated these changes. A randomized controlled trial should be conducted in sHT patients with different TSH levels to confirm these findings. The impact of LT_4_ treatment on other AS risk factors also should be explored. Further mechanisms should be investigated to explain the role of adipokines in AS in the context of sHT.

## Declaration of interest

The authors declare that there is no conflict of interest that could be perceived as prejudicing the impartiality of the research reported.

## Funding

This work was supported by the National Natural Science Foundation of China (grant number: 81360125) and Gansu Province Natural Science Fund Project (grant number: 17JR5RA041).
