# Adipokine expression and endothelial function in subclinical hypothyroidism rats

**DOI:** 10.1530/EC-18-0007e

**Published:** 2018-04-01

**Authors:** Ningning Gong, Cuixia Gao, Xuedi Chen, Yu Wang, Limin Tian

**Affiliations:** 1 Department of Endocrinology, Gansu Provincial Hospital, Lanzhou, China; 2 Department of Clinical Medicine, Gansu University of Chinese Medicine, Lanzhou, Gansu, China; 3 Department of Ultrasonic Diagnosis, Gansu Provincial Hospital, Lanzhou, China

The authors and journal apologise for an error in the above paper, which appeared in volume 7 part 2, pages 295–304. The error relates to the text in the ‘Animal model’ section of the ‘Materials and methods’ section given on page 296:

The original article stated:

‘In the sHT rats, groups A–C were separately induced by administration of 20 mg/kg/day methimazole (MMI), 30 mg/kg/day MMI and 40 mg/kg/day MMI once daily by gavage.’

This should have stated:

‘In the sHT rats, groups A–C were separately induced by administration of 5 mg/kg/day methimazole (MMI), 15 mg/kg/day MMI and 20 mg/kg/day MMI once daily by gavage.’

